# Supplementary figures and images for: Magnesium Increases Homoeologous Crossover Frequency During Meiosis in ZIP4 (Ph1 Gene) Mutant Wheat-Wild Relative Hybrids
Source: Front Plant Sci. 2018 Apr 20;9:509. doi: 10.3389/fpls.2018.00509 (PMC5920029; doi:10.3389/fpls.2018.00509)

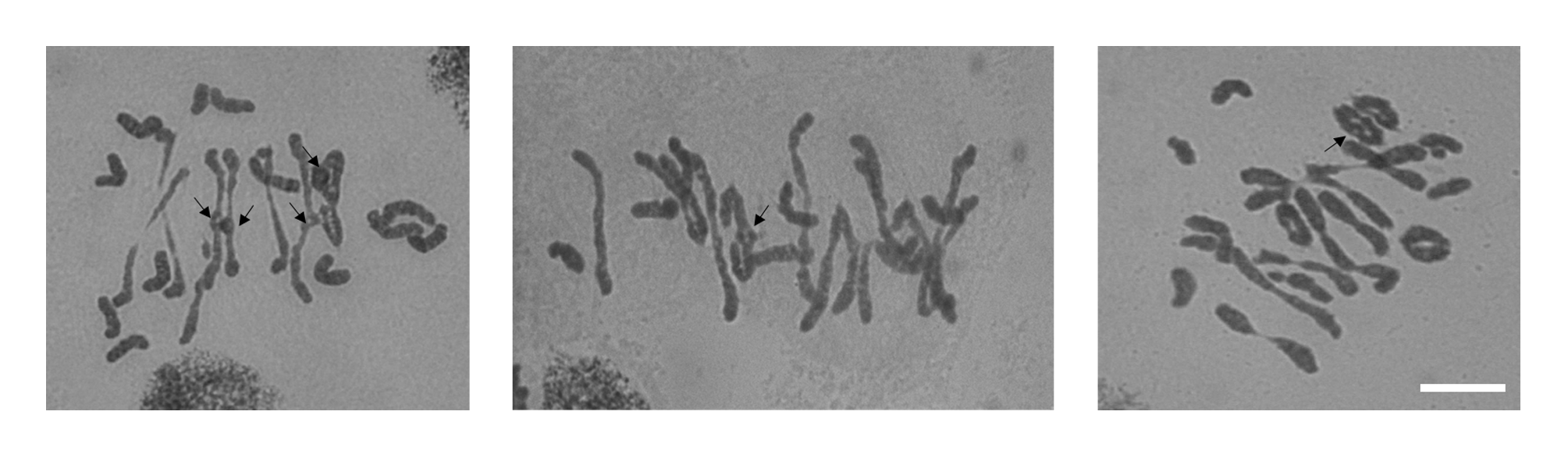

Supplement: Figure S1 — Chromosomal configurations with single chiasma or double chiasmata highlighted with arrows. These structures marked by an arrow were counted as either single or double chiasmata in all analyzed meiocytes. Both datasets are shown in all analyzed genotypes. Bar: 20 μm. [file Image1.TIF]

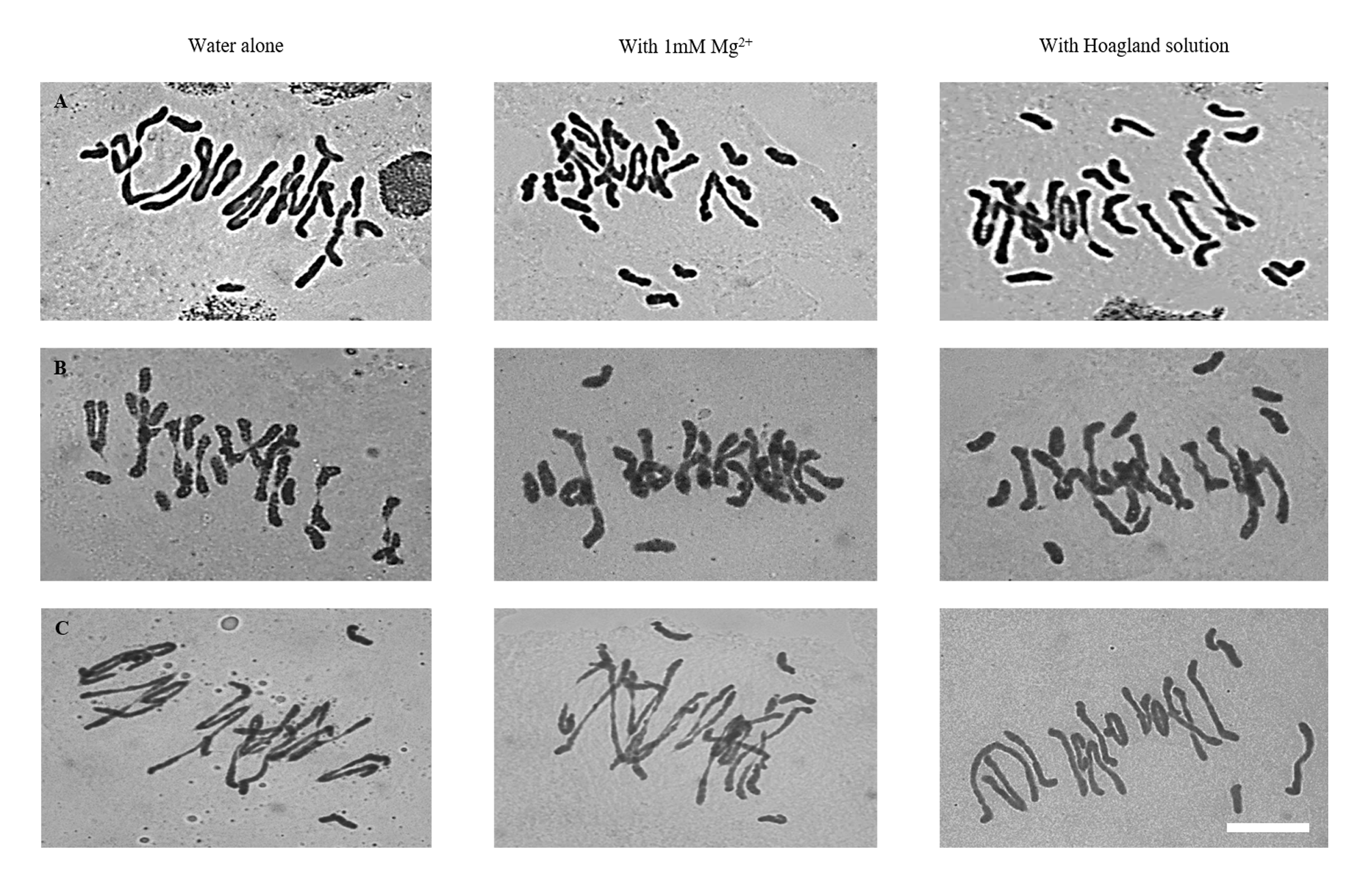

Supplement: Figure S2 — Representative meiotic configurations of Triticum aestivum cv. Cadenza (Cad1691-Tazip4-B2 mutant)-Ae. variabilis (A) and Triticum aestivum cv. Cadenza (Cad0348-Tazip4-B2 mutant)-Ae. variabilis (B) and wheat Tazip4-B2 CRISPR mutant-Ae. variabilis mutant (C) hybrids. From left to right: water alone, treated with either 1 mM Mg2+ or Hoagland solution. Bar: 20 μm. [file Image2.TIF]
