# Supplementary material for: Magnesium Increases Homoeologous Crossover Frequency During Meiosis in ZIP4 (Ph1 Gene) Mutant Wheat-Wild Relative Hybrids
Source: Front Plant Sci. 2018 Apr 20;9:509. doi: 10.3389/fpls.2018.00509 (PMC5920029; doi:10.3389/fpls.2018.00509)
Supplement: Table S1 — Genotypes and number of plants used for analyzing the effect of a nutrient solution in homoeologous CO frequency in wheat and its relative species. [file Table1.DOCX]

**Supporting information**

**TABLE S1 | Genotypes and number of plants used for analysing the effect of a nutrient solution in homoeologous CO frequency in wheat and its relative species**.

| **Genotype** | **Treatment** | **No. of plants** |
| --- | --- | --- |
| **Absence of the *Ph1* locus** |  |  |
| CS- x Rye hybrids | Hoagland Solution | 5 |
| CS- x Rye hybrids | without Hoagland | 5 |
| CS- x Rye hybrids | with Hoagland Solution - NH_2_ H_2_PO_4_ | 5 |
| CS- x Rye hybrids | with Hoagland Solution - KNO_3_ | 5 |
| CS- x Rye hybrids | with Hoagland Solution - CaNO_3_ | 5 |
| CS- x Rye hybrids | with Hoagland Solution - MgSO_4_ | 5 |
| CS- x Rye hybrids | with Hoagland | 5 |
| CS- x Rye hybrids | 1mM Magnesium | 5 |
| CS- x Rye hybrids | 2mM Magnesium | 5 |
| CS- x *Ae. variabilis* hybrids | without 1mM Magnesium | 4 |
| CS- x *Ae. variabilis* hybrids | 1mM Magnesium | 5 |
| CS- x *Ae. variabilis* hybrids | Hoagland Solution | 3 |
| **Absence of the *TaZIP4* gene** |  |  |
| **TILLING** |  |  |
| Cad1691 x *Ae. variabilis* hybrids | without 1mM Magnesium | 5 |
| Cad1691 x *Ae. variabilis* hybrids | 1mM Magnesium | 4 |
| Cad1691 x *Ae. variabilis* hybrids | Hoagland Solution | 4 |
| Cad0348 x *Ae. variabilis* hybrids | without 1mM Magnesium | 5 |
| Cad0348 x *Ae. variabilis* hybrids | 1mM Magnesium | 4 |
| Cad0348 x *Ae. variabilis* hybrids | Hoagland Solution | 4 |
| **CRISPR/Cas9 system** |  |  |
| Wheat cv. Fielder carrying *TaZIP4-B2* |  | 5 |
| Wheat cv. Fielder lacking *TaZIP4-B2* |  | 5 |
| *TaZIP4-B2* - *Ae. variabilis hybrids* |  | 4 |
| *Tazip4-B2* CRISPR - *Ae. variabilis hybrids* | without 1mM Magnesium | 4 |
| *Tazip4-B2* CRISPR - *Ae. variabilis hybrids* | 1mM Magnesium | 4 |
| *Tazip4-B2* CRISPR - *Ae. variabilis hybrids* | Hoagland Solution | 4 |
