# Supplementary material for: Magnesium Increases Homoeologous Crossover Frequency During Meiosis in ZIP4 (Ph1 Gene) Mutant Wheat-Wild Relative Hybrids
Source: Front Plant Sci. 2018 Apr 20;9:509. doi: 10.3389/fpls.2018.00509 (PMC5920029; doi:10.3389/fpls.2018.00509)
Supplement: Table S2 — Frequencies of univalents, bivalents, multivalents and chiasma frequency (single and double chiasmata) were scored at meiotic metaphase I in wheat Tazip4-B2 CRISPR mutant-Ae. variabilis hybrids. Values in parenthesis indicate range of variation between cells. P < 0.05 indicates significant differences according to Dunn's test. Different letters indicate significant differences between treatments. [file Table2.DOCX]

**Supporting information**

**TABLE S2 |** **Frequencies of univalents, bivalents, multivalents and chiasma frequency (single and double chiasmata) were scored at meiotic metaphase I in wheat *Tazip4-B2* CRISPR mutant - *Ae. variabilis* hybrids.** Values in parenthesis indicate range of variation between cells. P < 0.05 indicates significant differences according to Dunn´s test.

|  | **No. of cell examined** | **Univalents** | **Rod bivalents** | **Ring bivalents** | **Trivalents** | **Tetravalents** | **Pentavalents** | **Chiasma frequency** | |
| --- | --- | --- | --- | --- | --- | --- | --- | --- | --- |
|  |  | **Mean ± SE (Range)** | **Mean ± SE (Range)** | **Mean ± SE (Range)** | **Mean ± SE (Range)** | **Mean ± SE (Range)** | **Mean ± SE (Range)** | **Mean ± SE (Range)** | **Mean ± SE**  **(Range)** |
|  |  |  |  |  |  |  |  | **Single Chiasma** | **Double Chiasmata** |
| **Fielder x *Ae. variabilis* hybrids** | 172 | 28.99 ± 0.27^a^  (20-35) | 2.61 ± 0.12^b^  (0-7) | 0.05 ± 0.02^b^  (0-1) | 0.23 ± 0.04^b^  (0-2) | - | - | 3.15 ± 0.15^b^  (0-8) | 3.41 ± 0.17^b^  (0-7) |
| **CRISPR x *Ae. variabilis* hybrids** | 124 | 9.64 ± 0.27^b^  (3-17) | 5.64 ± 0.17^a^  (2-10) | 1.94 ± 0.11^a^  (0-6) | 2.37 ± 0.11^a^  (0-6) | 0.52 ± 0.06  (0-3) | 0.20 ± 0.04  (0-2) | 16.66 ± 0.21^a^  (11-22) | 18.10 ± 0.23^a^  (12-24) |
| ***P-value*** |  | *0.0000* | *0.0000* | *0.0000* | *0.0000* | *-* | *-* | *0.0000* | *0.0000* |
